# Pittsburgh compound B (PiB) PET imaging of meningioma and other intracranial tumors

**DOI:** 10.1007/s11060-017-2661-z

**Published:** 2017-11-08

**Authors:** Derek R. Johnson, Christopher H. Hunt, Mark A. Nathan, Joseph E. Parisi, Bradley F. Boeve, Melissa E. Murray, David S. Knopman, Clifford R. Jack, Ronald C. Petersen, Val J. Lowe, Geoffrey B. Johnson

**Affiliations:** 1 Department of Radiology, Mayo Clinic, 200 First St SW, Rochester, MN 55905 USA; 2 Department of Laboratory Medicine and Pathology, Mayo Clinic, Rochester, MN USA; 3 Department of Neurology, Mayo Clinic, Rochester, MN USA; 4 Department of Neuroscience, Mayo Clinic, Jacksonville, FL USA

**Keywords:** Meningioma, Beta-amyloid, Positron emission tomography (PET), PiB, Metastasis

## Abstract

Meningiomas are the most common intracranial tumors. Diagnosis by MRI is generally straightforward, but lack of imaging specificity can present a diagnostic dilemma, particularly in patients with cancer. We report our experience with meningioma identification on Pittsburgh compound B (PiB) PET/CT. Patients who underwent PiB PET/CT from 2006 to 2015 were reviewed to identify those with intracranial tumors. Tumor types were classified by MR appearance, or by pathology when available. Maximum standardized uptake value (SUVmax) measurements of tumor PiB activity were compared across tumor types. 2472 patients underwent PiB PET/CT in the period of interest; 45 patients (1.8%) had probable or definite intracranial tumor. Tumor types were meningioma (29/45, 64%), vestibular schwannoma (7/45, 16%), pituitary macroadenoma (4/45, 9%), metastatic disease (2/45, 4%), and others (3/45, 7%). In patients with meningioma, the mean lesion SUVmax was 2.05 (SD 1.37), versus 1.00 (SD 0.42) in patients with non-meningioma tumors (p < 0.01). A receiver operating curve was created for lesion:cerebellum SUVmax ratio, with an area under the curve of 0.91 for a value of 1.68. At or above this ratio, specificity for meningioma was 100% (95% CI 79–100%) and sensitivity was 76% (95% CI 57–90%). PiB PET activity within an intracranial tumor is a highly specific and reasonably sensitive marker of meningioma. Further prospective evaluation is warranted to validate this result as well as to assess the performance of commercially available beta-amyloid radiotracers in meningioma identification.

## Introduction

Meningiomas are the most common intracranial tumor in adults. Although diagnosis based on neuroimaging usually is straightforward, this is not the case in patients with a history of cancer, in whom the identification of a dural-based mass raises the question of metastasis, with profound implications for treatment and prognosis. As there are currently over 14 million cancer survivors in the United States, and the prevalence of meningioma in adults is approximately 0.9–3%, [1] this is a common clinical scenario. Meningeal metastases are also relatively common, with an incidence of 9–10% in patients with late-stage cancer [2, 3]. Meningeal metastases are not limited to patients with widely metastatic disease; approximately 20% of patients have an otherwise limited and potentially curable stage of disease or have previously controlled cancer elsewhere at the time of diagnosis [2]. A non-invasive imaging test with the ability to definitively identify meningioma would thus have the potential to significantly improve patient management.

Currently available imaging techniques such as CT and MRI offer limited specificity for differentiating meningioma from other meningeal lesions. The “dural tail” often considered characteristic of meningioma occurs in other conditions as well, [4–7] including approximately 44% of dural metastases [2]. As a result of the limited specificity of imaging, neurosurgical intervention usually is required for definitive diagnosis, with associated costs and morbidity. Occasionally patients are treated empirically with radiation, but this approach also carries potential morbidity and leaves the diagnosis unresolved. Alternatively, an observational approach may be taken, but in cases that prove to be metastasis this delays appropriate therapy.

Pittsburgh complex B (PiB) is a benzothiazole derivative developed as a positron emission tomography (PET) imaging agent, specifically designed to bind to beta-amyloid plaques in the brains of patients with Alzheimer’s disease [8]. Since we first observed the localization of PiB to meningiomas, several cases with this finding have been reported [9–11]. We reviewed our institutional experience with PiB PET/CT to evaluate its utility in identifying meningiomas, as well as to describe the PiB PET characteristics of other incidentally discovered intracranial tumors.

## Materials and methods

Our internal review board approved this research in advance. Medical records of all patients who underwent PiB PET/CT of the brain at Mayo Clinic in Rochester, MN from March 2006 through December 2015 were reviewed to identify individuals with intracranial tumors. The vast majority of these patients were participants in the Mayo Clinic Study of Aging (MCSA), which also included at least one non-contrast MRI [12]. Many of the patients had also undergone gadolinium-enhanced MRI of the brain, either for clinical purposes unrelated to this study or after identification of suspicious lesions on study imaging.

Tumors identified on MRI were categorized as probable meningiomas or non-meningiomas based on the assessment of the reporting board-certified neuroradiologist. Tumors classified as probable meningiomas were further reviewed, and included in this analysis if the diagnosis was pathologically confirmed or if the tumor was shown to be enhancing on post-gadolinium MRI, to exclude benign dural calcifications, and if the subject had at least 6 months of follow-up imaging confirming stability to exclude metastases. When intracranial metastatic disease was suspected, the diagnosis was confirmed by surgery. Tumors that were not consistent with meningioma or metastatic disease were categorized based on MRI appearance, location, and pathology when available. Tumors smaller than 5 mm in shortest diameter were excluded from analysis, given that they fall below the generally accepted lower limits of PET/CT resolution.

[C-11] PiB PET/CT imaging was performed on a Discovery 690XT or RX PET/CT tomograph (GE Healthcare; Waukesha, WI). CT imaging was obtained immediately prior to PET acquisition and used for attenuation correction. [C-11] PiB was produced on-site in our cyclotron facility. Production and quality control methods are described elsewhere [13]. PiB PET/CT images were reviewed to identify the tumors discovered on MRI. Maximum standardized uptake value (SUVmax) measurements and image fusion were performed using OsiriX Open-Source PACS Workstation, 64-bit version 7.5 (Pixmeo; Geneva, Switzerland). SUVmax also was measured in the lateral cerebellar hemisphere as an internal control, and the tumor/cerebellum SUVmax ratio was calculated for each patient. The cerebellum was chosen since it is considered to have consistent background activity and is not a site where cerebral beta-amyloid deposition generally occurs in patients with preclinical Alzheimer’s disease.

Unpaired *t*-tests and one-way ANOVA were used to evaluate differences in continuous variables between two or more than two groups, respectively. The chi-squared test was used for categorical analyses. Two-tailed statistical tests were used to compute p-values. These analyses, as well as sensitivity/specificity analysis and ROC-curve creation were performed in JMP Pro 12.2.0 (SAS Institute Inc.; Cary, NC).

## Results

2472 patients underwent PiB PET/CT during the period of interest. 45 patients (1.8%) were identified with probable or definite intracranial tumor. Of these 45 patients, 29 (64%) had definite or probable meningiomas, and 16 (36%) had other tumor types including vestibular schwannoma (7/45, 16%), pituitary macroadenoma (4/45, 9%), metastatic disease (2/45, 4%), intraventricular tumors (2/45, 4%) and epidermoid (1/45, 2%).

Median age of the 45 patients with intracranial tumors was 74 years (range 51–95). The patients with meningioma were older (median age 76 years, range 52–95) than the patients with other tumors (median age 71.5 years, range 51–87; p = 0.05). Overall, women represented 58% (26/45) of patients with tumors. Of the patients with meningioma, 69% (20/29) were women, while only 38% (6/16) of patients with other tumor types were women; p = 0.04.

Of the 29 patients with definite or probable meningiomas radiographically, two tumors (7%) were pathologically confirmed to represent WHO grade I meningiomas. The remaining 27 (93%) were categorized based on MRI appearance, contrast enhancement, and stability over time. The largest proportion of patients with meningioma (14/29; 48%) had tumor overlying the cerebral convexities or involving the falx, while 7/29 (24%) had supratentorial skull base tumors and 8/29 (28%) had infratentorial or tentorium-based meningiomas. Tumor thickness ranged from 0.5 to 3.1 cm, with a median thickness of 1.2 cm. Eight tumors (28%) demonstrated at least partial calcification.

**Fig. 1:**
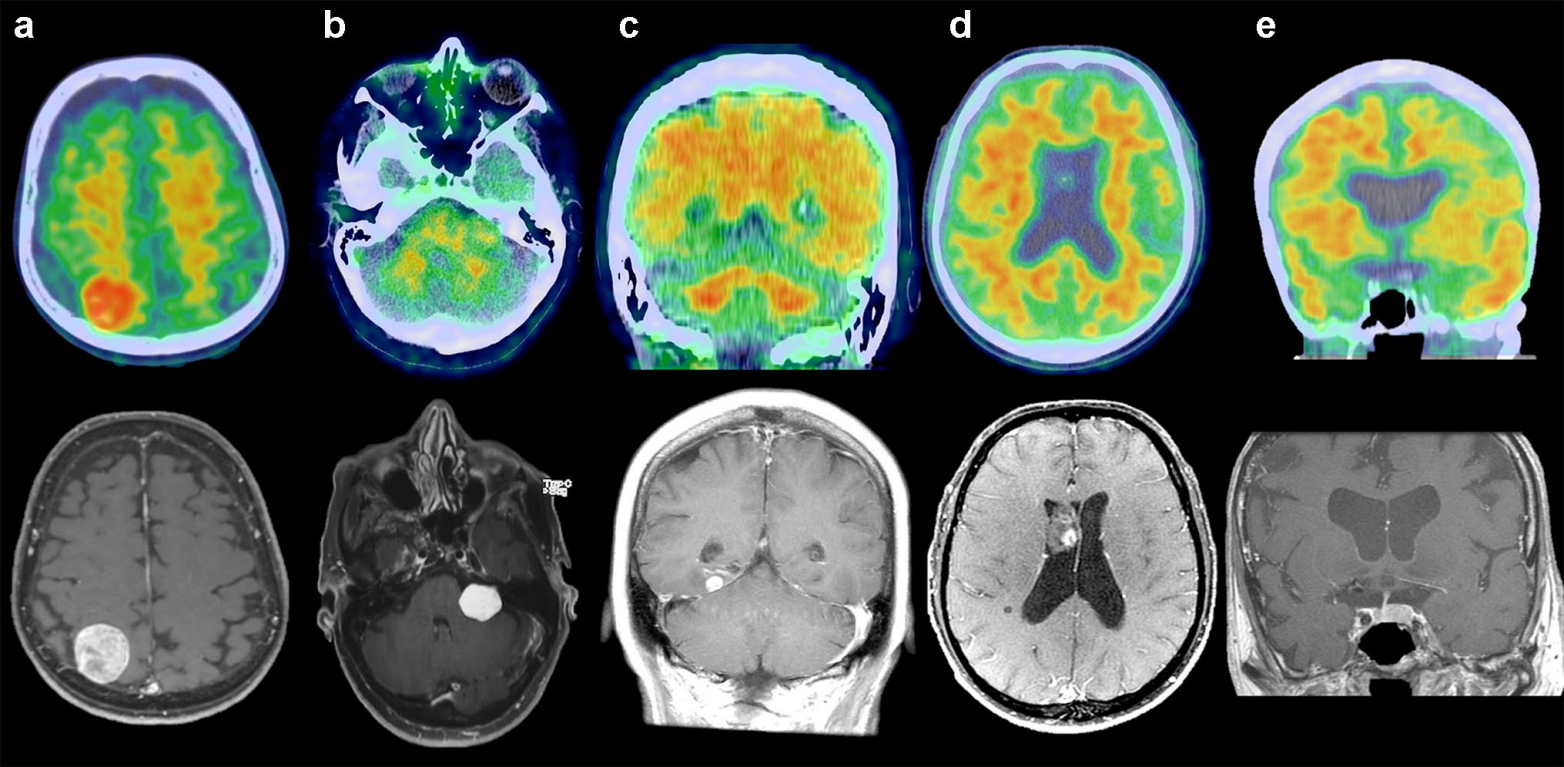
Examples of PiB PET/CT (top row) and T1-weighted post-contrast MRI (bottom row) appearance of meningioma (**a**), vestibular schwannoma (**b**), metastatic disease (**c**), central neurocytoma (**d**), and pituitary macroadenoma (**e**). Intense PiB activity is seen only in the meningioma (**a**)

**Fig. 2:**
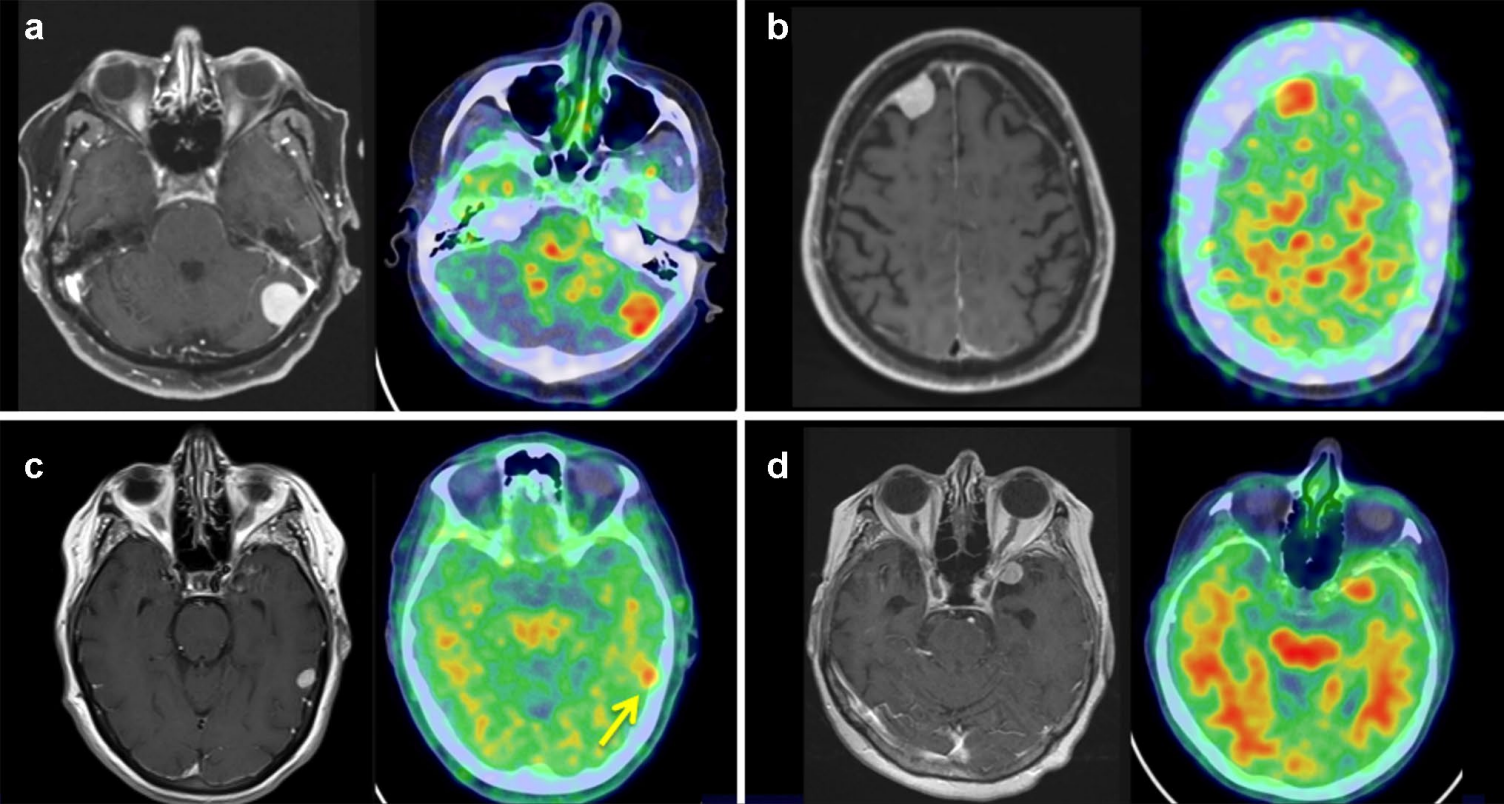
Additional meningioma PiB PET/CT images demonstrating PiB uptake in tumors abutting the left cerebellar hemisphere (**a**), right frontal lobe (**b**), posterior left temporal lobe (**c**), and left anteromedial temporal lobe (**d**), with companion T1-weighted post-contrast MR images

In patients with meningioma, the mean lesion SUVmax was 2.05 (SD 1.37), versus 1.00 (SD 0.42) in patients with non-meningioma tumors (p < 0.01). The mean lesion:cerebellum SUVmax ratio in meningioma was 2.12 (SD 0.70) versus 1.10 (SD 0.30) for non-meningioma tumors (p < 0.01). Figure 1 demonstrates the PiB PET/CT appearance of five different types of intracranial tumors, Fig. 2 displays several examples of meningiomas on PiB PET/CT. Meningiomas without internal calcification demonstrated a higher mean tumor:cerebellum SUVmax ratio of 2.32 compared to calcified tumors (mean ratio 1.59; p < 0.01). Absolute lesion SUVmax and lesion:cerebellum ratios were also both positively correlated with tumor size (p < 0.01).

Of the 16 patients with non-meningioma tumors, the two patients with metastatic disease (melanoma, small cell lung cancer) and two of the seven patients with vestibular schwannoma had pathologic confirmation of tumor type; the rest were categorized based on MR imaging appearance. Mean SUVmax did not vary significantly amongst the non-meningioma tumors (p = 0.31), ranging from a low of 0.80 (SD 0.39) for the seven vestibular schwannomas to a high of 1.4 (SD 0.72) for the two metastases. The mean lesion:cerebellum SUVmax ratio likewise did not differ amongst the non-meningioma tumor types (p = 0.50). Ratio values ranged from 0.96 (SD 0.35) for vestibular schwannoma to 1.39 (SD 0.24) for the two intraventricular tumors. Absolute SUVmax values and tumor:cerebellum SUV max ratios for each tumor type are displayed in Table 1.

**Table 1:** PiB PET activity by tumor type

		Lesion SUVmax		Lesion:cerebellum SUVmax ratio	
Tumor type	N	Mean	SD	Mean	SD
Meningioma	29	2.05	1.37	2.12	0.70
Vestibular schwannoma	7	0.80	0.39	0.96	0.35
Pituitary macroadenoma	4	0.94	0.19	1.19	0.26
Metastasis	2	1.40	0.72	1.13	0.24
Intraventricular mass	2	1.37	0.54	1.39	0.29
Epidermoid	1	1.13	–	1.06	–

**Fig. 3:**
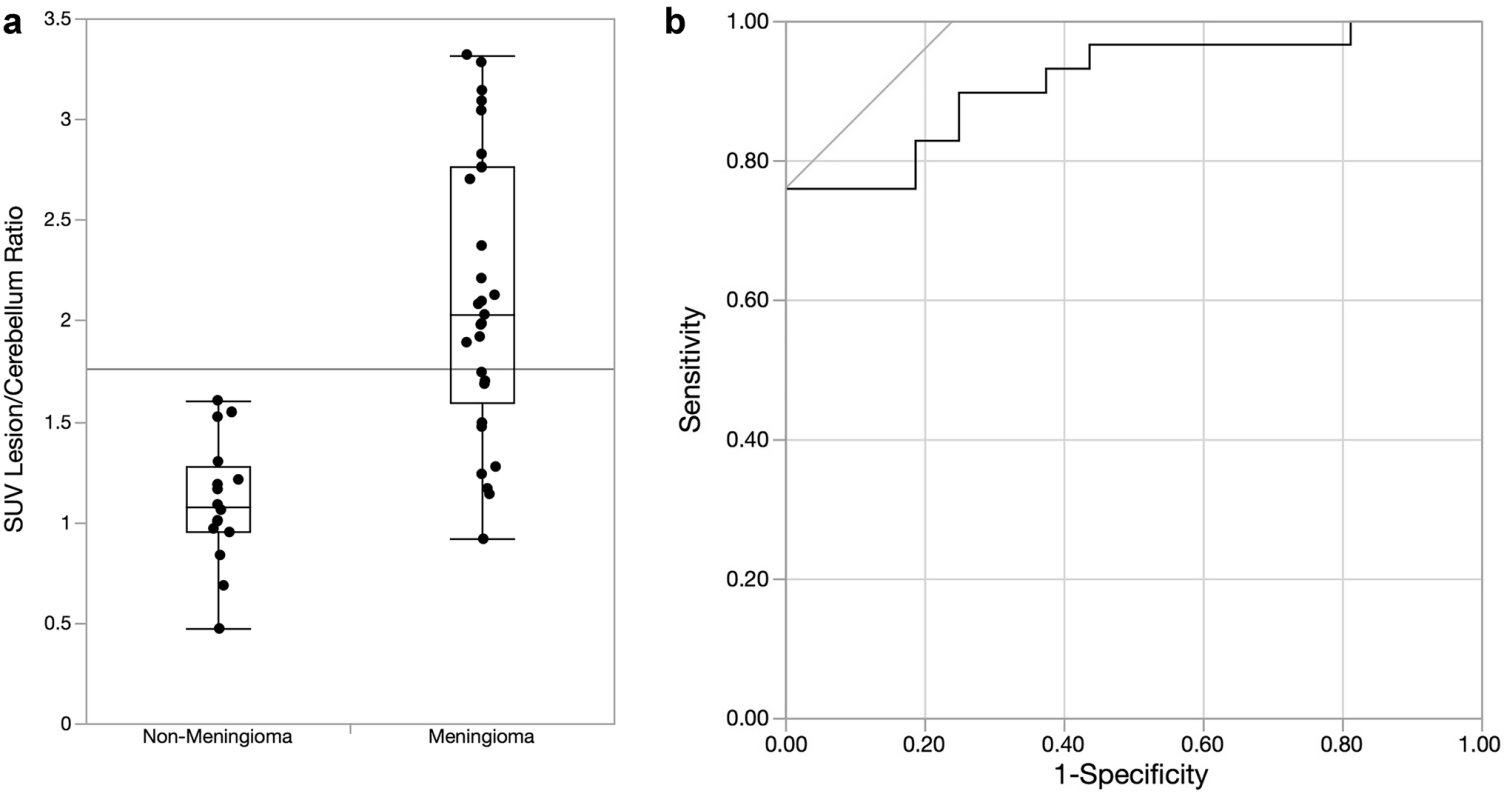
Quantile plot of tumor to cerebellum SUV ratios for meningioma and non-meningioma tumors (**a**) and the receiver-operating curve (ROC) for the test, with a ratio cutoff of **1.68** (**b**)

An ROC curve (Fig. 3) was created for the lesion:cerebellum SUVmax ratio, with an AUC of 0.91 for the ratio value of 1.68. At or above this ratio, specificity for meningioma was 100% (95% CI 79–100%) and sensitivity was 76% (95% CI 57–90%). The positive predictive value was 100% (95% CI 85–100%).

Amongst the 29 patients with meningioma, those with larger tumors were more likely to demonstrate a PiB PET SUVmax lesion:cerebellum ratio of 1.68 or above (p < 0.01). The mean thickness of tumors with an SUVmax ratio of 1.68 or above was 1.58 cm, versus a median thickness of 0.79 cm for meningiomas below this threshold. Calcified tumors were less likely to demonstrate high-level PiB uptake (p < 0.01), with only 3 of 8 (37.5%) calcified meningiomas demonstrating an SUVmax ratio of 1.68 or above, compared to 19 of 21 (90.5%) of non-calcified meningiomas. There was no association between SUVmax ratio and either patient age or sex (each p > 0.05).

## Discussion

This retrospective analysis suggests that PiB PET/CT can identify meningiomas 5 mm or greater in size with fair sensitivity and differentiate meningioma from other intracranial tumors with excellent specificity. Thus, PiB PET/CT may help resolve a common dilemma in brain tumor diagnosis.

An imaging test with the ability to definitively differentiate meningioma from other intracranial tumors would be extremely beneficial to patients. In patients receiving initial staging for cancer in whom a dural-based mass is the only potential distant metastasis, PiB PET/CT may be the difference between offering treatment with curative or palliative intent. If the PiB PET/CT confirms the diagnosis of meningioma, aggressive therapy with curative intent can be offered for the primary tumor without the delay, cost, or morbidity of needle biopsy or craniotomy for mass resection. Conversely, if PiB PET/CT indicates that meningioma is highly unlikely, treatment for metastasis can be offered immediately, preventing further tumor growth and associated neurological symptoms.

A number of other PET and SPECT tracers may have roles in meningioma imaging, but they lack the specificity of PiB. FDG is by far the most commonly used PET tracer in cancer, but it has limited utility in brain tumor imaging generally or meningioma specifically because of physiological high FDG activity within the brain and lack of specificity. Agents targeting somatostatin receptors, such as the SPECT agent indium-111 entetreotide (Octreoscan™) and the PET radiopharmaceutical Gallium-68 DOTATATE (NETSPOT™) differentiate meningioma from the surrounding brain, but lack specificity given the presence of somatostatin receptors in other primary CNS tumors [14] and brain metastases of neuroendocrine tumors. C11-choline is a PET tracer most commonly used in prostate cancer that also has activity in a variety of other tumors, including meningioma [15, 16]. While one or more of these tracers may ultimately prove useful for meningioma surveillance or response assessment, our analysis suggests that the high specificity of PiB for meningioma may give it a unique role in diagnosis.

While this analysis is by far the largest report of PiB PET findings in patients with intracranial masses, and the first evaluation of the sensitivity and specificity of this tool, additional studies will be necessary in order to translate these findings into clinical practice. First, this was a retrospective analysis that included numerous meningiomas but only a small number of each of the individual non-meningioma tumors. Relatively high lesion:cerebellum PiB PET SUVmax ratios were observed in the vast majority of meningiomas, particularly larger non-calcified meningiomas, demonstrating that this technique demonstrates good sensitivity for meningioma. However, if this technique is to be useful clinically to guide treatment decisions, high specificity will be more important than sensitivity. We demonstrate extremely high specificity in this study, but the analyses are based on comparison of meningioma PiB uptake to that of a heterogeneous collection of intracranial tumors. While this illustrates that PiB PET uptake is not a common feature of non-meningioma intracranial tumors, the specificity observed in this analysis should be confirmed in prospective studies involving a greater numbers of dural metastases, as differentiation of meningioma from metastasis is potentially the most useful application of this technique. Further, all of the meningiomas identified on PiB/PET were either proven to be WHO grade I on pathology or can be inferred to be low-grade by virtue of radiographic stability, so additional evaluation of WHO grade II and III meningiomas is warranted.

Our analysis utilized PiB, a beta-amyloid radiotracer widely used in research which is not FDA approved for clinical use and which requires an on-site cyclotron for production due to its short half-life. Several beta-amyloid PET tracers have been approved by the FDA, including florbetapir, florbetaben, and flutemetamol, with varying degrees of homology to PiB. It is not clear whether any or all of these compounds also demonstrate affinity for meningiomas. Future studies evaluating the diagnostic performance of these tracers for meningioma identification will be necessary, and may allow this technique to be used in a larger number of centers.

The affinity of PiB, and potentially other of amyloid PET agents, for both meningiomas and beta-amyloid plaques may present issues in image interpretation. The normal pattern of PiB uptake in patients without cerebral beta-amyloid deposition is nonspecific white matter uptake with sparing of the gray matter. As such, PiB uptake in meningiomas stands out from adjacent gray matter. In patients with Alzheimer’s disease, cortical tracer localization may decrease the conspicuity of small meningiomas. Conversely, the presence of a meningioma should not confound the assessment of Alzheimer’s disease, which is characterized by diffuse cortical uptake and loss of the normal gray/white differentiation on PiB PET. However, a meningioma could theoretically lead to erroneous quantification of gray matter PiB activity if inadvertently included in an SUV region of interest.

The biological/molecular basis for the binding of PiB to meningioma tissue has yet to be determined. The leptomeninges have been shown to contain deposits of beta-amyloid in both patients with Alzheimer disease and normal controls [17]. Some studies have suggested that subsets of meningiomas contain beta-amyloid as well [18, 19]. However, other studies examining the issue have suggested that beta-amyloid is not present in meningiomas [20]. In this analysis, the large majority of meningiomas, particularly non-calcified meningiomas, demonstrated PiB PET uptake greater than that of the cerebellar white matter. As our observed proportion of PiB-avid tumors is greater than the proportion of meningiomas suggested to contain beta-amyloid in any of the previous pathological analyses, the authors favor the possibility of a PiB binding target other than beta-amyloid within these tumors. While knowledge of the specific target of PiB is not mandatory for this technique to prove useful in meningioma diagnostic imaging, it does have relevance for theoretical future applications such as design of targeted therapies, and further investigation is warranted.

In conclusion, this study suggests that the radiotracer PiB has fairly specific affinity for meningiomas. This finding could potentially have numerous clinical implications, including but not limited to the utility of PiB PET/CT to differentiate meningioma from metastatic cancer or cerebellopontine angle meningioma from vestibular schwannoma.
